# Supplementary material for: Identification of photoperiod-induced specific miRNAs in the adrenal glands of Sunite sheep (Ovis aries)
Source: Front Vet Sci. 2022 Jul 22;9:888207. doi: 10.3389/fvets.2022.888207 (PMC9354845; doi:10.3389/fvets.2022.888207)
Supplement: Supplementary file 1 [file Data_Sheet_1.ZIP › Supplementary file/miRDeep2 file.zip!/miRDeep2 file/Novel_Structure/LP42A1/Novel_190.pdf]

Provisional ID	:	NC_019475.2_27337
Score total	:	125.6
Score for star read(s)	:	3.9
Score for read counts	:	117.9
Score for mfe	:	2.2
Score for randfold	:	1.6
Score for cons. seed	:	
Total read count	:	243
Mature read count	:	229
Loop read count	:	0
Star read count	:	14

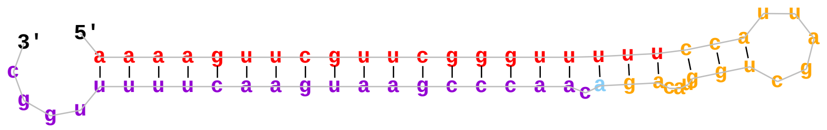

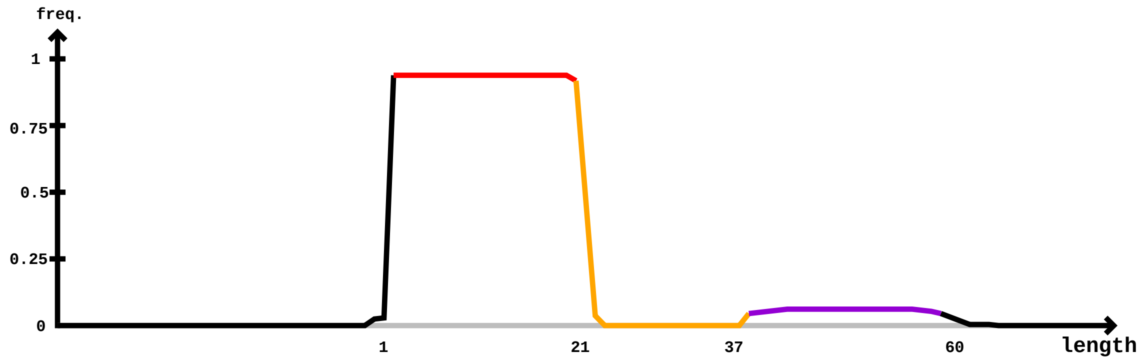

Mature Star

```
5'- ccugggggaggaagggauguacuggguuggcccaaaaguucguucggguuuuuccauuagcugguacagacaacccgaaugaacuuuuuggccaauucaauauuucca  -3'  obs
    ccugggggaggaagggauguacuggguuggcccaaaaguucguucggguuuuuccauuagcugguacagacaacccgaaugaacuuuuuggccaauucaauauuucca       exp
    (((.....)))...(((.(((.((((((((((.(((((((((((((((((((((((.....)))...))).))))))))))))))))).)))))))))).))).))).   reads   mm   sample
    ...............................cAaaaaguucguucggguuuuu.......................................................   6       1    seq
    ................................Aaaaaguucguucggguuuuuc......................................................   1       1    seq
    .................................aaaaguucguucggguuuu........................................................   5       0    seq
    .................................aaaaguucguucggguuuuC.......................................................   15      1    seq
    .................................aaaaguucguucggguuuuu.......................................................   108     0    seq
    .................................aaaaguucguucggguuuuCc......................................................   1       1    seq
    .................................aaaaguucguucggguuuuuc......................................................   84      0    seq
    .................................aaaaguucguucggguuuuucc.....................................................   3       0    seq
    .................................aaaaguucguucggguuuuucU.....................................................   6       1    seq
    ......................................................................caacccgaaugaacuuuu....................   1       0    seq
    ......................................................................caacccgaaugaacuuuuug..................   2       0    seq
    ......................................................................caacccgaaugaacuuuuugg.................   1       0    seq
    ......................................................................Aaacccgaaugaacuuuuuggc................   7       1    seq
    .......................................................................aacccgaaugaacuuuuu...................   1       0    seq
    ........................................................................acccgaaugaacuuuuuggcU...............   1       1    seq
    .........................................................................cccgaaugaacuuuuuggcA...............   1       1    seq
    ..........................................................................ccgaaCgaacuuuuuggccaau............   1       1    seq
```
